# PP61 Equity As A Criterion For Prioritizing Topics For The Development Of Clinical Practice Guidelines

**DOI:** 10.1017/S0266462325102808

**Published:** 2025-12-29

**Authors:** Maíra Pereira, Adriane Lopes Medeiros Simone, Tainá Saldanha, Mônica Rodrigues, Tamiê Martins, Daniela Melo

## Abstract

**Introduction:**

Equity is a pivotal element in health technology assessment (HTA) to ensure fair and proportional access to healthcare needs and to mitigate health disparities. The incorporation of equity-oriented prioritization criteria into the development of clinical practice guidelines (CPGs) promotes social justice in health. This study systematically mapped equity-related criteria used to prioritize topics for CPGs.

**Methods:**

A comprehensive scoping review was conducted in accordance with the Joanna Briggs Institute’ Manual for Evidence Synthesis and the PRISMA Extension for Scoping Reviews. The review protocol was pre-registered on the Open Science Framework. The review encompassed both peer-reviewed publications and gray literature published from 2000 onwards, without language limitations, that addressed topic prioritization criteria for CPG development. From an initial list of 22,675 records, 25 studies met the inclusion criteria. The extracted prioritization criteria were categorized into six domains and further refined into 20 distinct criterion.

**Results:**

Of the included studies, 48 percent mentioned the criterion “equity relevance”, within the domain of “factors related to the health condition”, and/or “impact on equity/access” within the domain of “potential impact of the intervention”. These criteria were variably characterized, encompassing aspects such as “frequency of inequality-related issues in specific population groups (e.g., children under five years old)”, “inequities in access and specific needs of vulnerable groups”, “potential of the recommendation to reduce or increase health disparities”, “impact on patient autonomy and independence,” and “user perceptions of the CPG.”

**Conclusions:**

Evaluating equity considerations related to a disease or health condition and the potential of a CPG to mitigate disparities are imperative in the topic prioritization process. Incorporating this approach can optimize resource allocation, enhance care for marginalized groups, promote social justice, reduce health disparities, and foster social cohesion, thereby contributing to public health and societal well-being.

